# A pronociceptive role for the 5-HT2 receptor on spinal nociceptive transmission: An in vivo electrophysiological study in the rat

**DOI:** 10.1016/j.brainres.2011.01.057

**Published:** 2011-03-25

**Authors:** Wahida Rahman, Kirsty Bannister, Lucy A. Bee, Anthony H. Dickenson

**Affiliations:** Department of Neuroscience, Physiology and Pharmacology, University College London, Gower Street, London WC1E 6BT, UK

**Keywords:** Serotonergic, 5-hydroxytryptamine2 receptor (5-HT2), Spinal cord, Wide dynamic range neurone, Nociceptive

## Abstract

Serotonin (5-HT) plays a major yet complex role in modulating spinal nociceptive transmission as a consequence of the number of 5-HT receptor subtypes. These include the 5-HT2 receptor, which is further sub classified into 5-HT2A, B and C. Studies have described both a pro- and antinociceptive action following 5-HT2A-receptor activation; therefore, to shed light on the directional nature of spinal 5-HT2A receptor activity, we investigated the effects of spinal administration of the 5-HT2A receptor antagonist, ketanserin, on the evoked responses of dorsal horn neurones to electrical, mechanical and thermal stimulation. We also assessed the effects of systemic administration of ritanserin, a 5-HT2A/2C receptor antagonist and spinal application of (±)-2,5-Dimethoxy-4-iodoamphetamine hydrochloride (DOI) (3.6 and 17.8 μg/50 μl), a 5-HT2A/2C agonist, on the same evoked neuronal responses. Ketanserin (1, 10 and 100 μg/50 μl) produced a dose related inhibition of the evoked responses to noxious mechanical punctate and thermal stimuli only. Ritanserin (2 mg/kg) replicated the inhibitory effects seen with ketanserin on the natural evoked neuronal responses and also potently inhibited the C-fibre, post discharge, input and wind-up evoked responses. DOI increased the mechanical and thermal evoked responses, an effect reversed by ketanserin. Thus, our findings show that spinal ketanserin (1–100 μg/50 μl) and systemic ritanserin (2 mg/kg), at these doses, have similar antinociceptive effects, whereas the agonist, DOI, produced excitatory effects, on spinal neuronal activity. Our data, therefore, supports a pronociceptive role for 5-HT2 receptors, most likely through modulation of 5-HT2A receptor activity, on spinal nociceptive transmission under normal conditions.

## Research Highlights

► Complex role for 5-HT2 receptor in spinal pain modulation. ► 5-HT2A receptor antagonist, ketanserin, inhibits evoked spinal neuronal responses. ► 5-HT2A/2C receptor antagonist, ritanserin, inhibits spinal neuronal responses. ► 5-HT2A/2C receptor agonist, DOI, increases evoked spinal neuronal responses. ► Inhibitory effects of ketanserin and ritanserin are comparable. ► 5-HT2A receptor has a pronociceptive role in spinal nociceptive transmission.

## 1 Introduction

Descending modulation from brainstem areas of spinal nociceptive transmission is a well-documented phenomenon. Most early studies describe a role for descending inhibitory control of spinal nociceptive activity mediated primarily by noradrenergic and serotonergic (5-HT) pathways, but more recently, the role of descending facilitation from the brainstem, onto spinal nociceptive pathways, has stimulated intense research and, in particular, the role for 5-HT in mediating this excitatory drive (Bannister et al., 2009; Wei et al., 2010).

Serotonergic input to the dorsal horn of the spinal cord derives almost entirely from supraspinal sources, with a minor contribution from local spinal neurones. 5-HT pathways, running directly from the rostral ventromedial medulla (RVM; the site of origin of the serotonergic descending pathway) to the spinal cord, comprise one of the main neurotransmitter systems mediating descending modulation of spinal neuronal activity. Animal studies report variably on the function of descending controls from the RVM and of 5-HT in nociceptive transmission (Bannister et al., 2009; Millan, 2002). Early studies investigating blockade of RVM activity and loss of 5-HT modulation have pointed to a loss of inhibitory control resulting in increased pain behaviours (Millan, 2002). However, in addition to descending inhibition, a wealth of evidence now exists for a descending excitatory drive from the RVM modulating spinal nociceptive transmission, which involves the activation of serotonergic pathways (Bannister et al., 2009; Dogrul et al., 2009; Wei et al., 2010).

The heterogeneous nature of the 5-HT receptor family underlies the bidirectional effect of the neurotransmitter. To date, seven different receptor subfamilies have been identified which vary with respect to their localisation, coupling and ligand binding properties (Alexander et al., 2008). A number of reports have linked descending facilitation from the brainstem to activation of spinal 5-HT3 receptors (Dogrul et al., 2009; Rygh et al., 2006; Suzuki et al., 2002; Svensson et al., 2006). For instance, using *in vivo* electrophysiological methods, we have demonstrated a pro-nociceptive function for spinal 5-HT3 receptors on spinal neuronal activity since topical spinal application of the selective antagonist ondansetron significantly reduced spinal neuronal activity in normal and pathaphysiological conditions (Rahman et al., 2004; Suzuki et al., 2002; Suzuki et al., 2004). This pronociceptive role for spinal 5-HT3 receptors has also been borne out by behavioural and anatomical studies (Dogrul et al., 2009; Oatway et al., 2004; Svensson et al., 2006; Zeitz et al., 2002) but disputed by others (Peters et al., 2010), although a pronociceptive role of endogenous spinal 5-HT was demonstrated by the reduction in nociceptive responses following selective depletion of spinal 5-HT (Dogrul et al., 2009; Oatway et al., 2004; Rahman et al., 2006). Nonetheless, descending serotonergic facilitation may not be exclusive to 5-HT activating the 5-HT3 receptor, as there are several lines of evidence pointing to a pronociceptive role for the 5-HT2 receptor, although controversy exists.

The complexity of effects produced by 5-HT acting on 5-HT2 receptors is due to the further existence of subtypes, namely 5-HT2A, 2B and 2C receptors (Alexander et al., 2008). Of these, the evidence to date largely points to a pronociceptive role for the 5-HT2A subtype (Eide and Hole, 1991; Kjorsvik et al., 2001; Nishiyama, 2005; Silveira et al., 2010; Thibault et al., 2008) but see (Honda et al., 2006; Kommalage and Hoglund, 2005; Sasaki et al., 2001; Sasaki et al., 2003), and an antinociceptive role for the 5-HT2C receptor subtypes in modulating spinal nociceptive transmission (Aira et al., 2010; Liu et al., 2007; Obata et al., 2004; Obata et al., 2007). The amino acid sequence of the 5-HT2 receptors share a high degree of homology within the seven transmembrane domains; thus, it is not surprising that conflicting reports exist within the literature since many compounds bind to each subtype with high affinity (Knight et al., 2004). Behavioural studies could be confounded by the multiple functions of 5-HT in the CNS. Here, we evaluate the effect of topical spinal application of the selective 5-HT2A receptor antagonist, ketanserin, on the evoked responses of wide dynamic range dorsal horn neurones in response to electrical and natural stimulation of the peripheral receptive field, in order to evaluate the spinal specific role of this receptor subtype in suprathreshold responses. Ketanserin potently blocks 5-HT2A receptors, less potently blocks 5-HT2C receptors, and has no significant effect on 5-HT3 or 5-HT4 receptors or any members of the 5-HT1 receptor family (Knight et al., 2004). We also assessed the effects of systemic delivery of the 5-HT2A/2C antagonist, ritanserin, on the same neuronal measures. Ritanserin has equal affinity for the 5-HT2A and 2C subtypes (Knight et al., 2004), and finally, we assessed the effects of spinal application of (±)-2,5-Dimethoxy-4-iodoamphetamine hydrochloride (DOI), a mixed 5-HT2A/2C agonist, but with greater relative selectivity for 5-HT2A receptors, on these evoked spinal neuronal responses.

## 2 Results

### 2.1 Effect of topical spinal administration of ketanserin on evoked dorsal horn neuronal responses

**Fig. 1:**
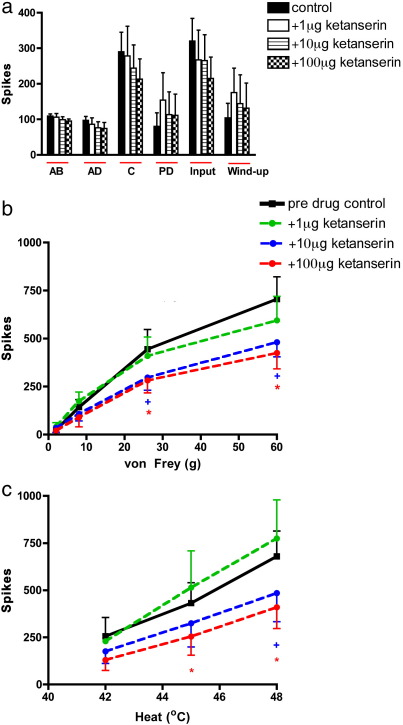
Effect of topical spinal administration of ketanserin (1, 10 and 100 μg/50 μl) on the a. electrical b. mechanical punctate and c. thermal evoked responses of wide dynamic range dorsal horn neurones. Ketanserin significantly inhibited the noxious mechanical (26 and 60 g) and heat (45–48 °C) evoked neuronal responses, evoked responses of wide dynamic range dorsal horn neurones. Data expressed as mean ± SEM. Significance at the lower dose of 10 μg/μl is denoted by +, whereas significance at the higher dose of 100 μg/μl is denoted by *p < 0.05.

Spinally applied ketanserin (1, 10 and 100 μg/50 μl) did not produce any significant effects on any of the electrically evoked neuronal measures, although a trend towards a dose-related inhibition was observed for the Aδ-, C-fibre and input evoked responses (Fig. 1a).

In contrast a significant dose-related inhibition was observed on the natural evoked neuronal responses. This was evidenced as the lower dose was ineffective and the two highest doses produced a significant inhibition of the noxious heat and mechanical punctate evoked neuronal responses (Fig. 1b and c).

Spinal application of cumulative doses of ketanserin inhibited neuronal responses to mechanical stimuli, seen as significant decreases in evoked neuronal response to stimulation with von Frey 26 and 60 g (significant at 10 μg and 100 μg, *p* < 0.05 2-way RM ANOVA). Significant inhibition of the evoked neuronal responses was also observed in response heat stimulation at 45 °C (significant 100 μg, *p* < 0.05 2-way RM ANOVA) and 48 °C (significant at 10 μg and 100 μg, *p* < 0.05 2-way RM ANOVA) (Fig. 1c).

Spinal application of ketanserin did not significantly inhibit any of the low-intensity innocuous mechanical (vF 2 and 8 g) and heat (35–40 °C) evoked responses nor the evoked response to noxious heat at 50 °C (Fig. 1b and c).

### 2.2 Effect of systemic administration of ritanserin on evoked dorsal horn neuronal responses

**Fig. 2:**
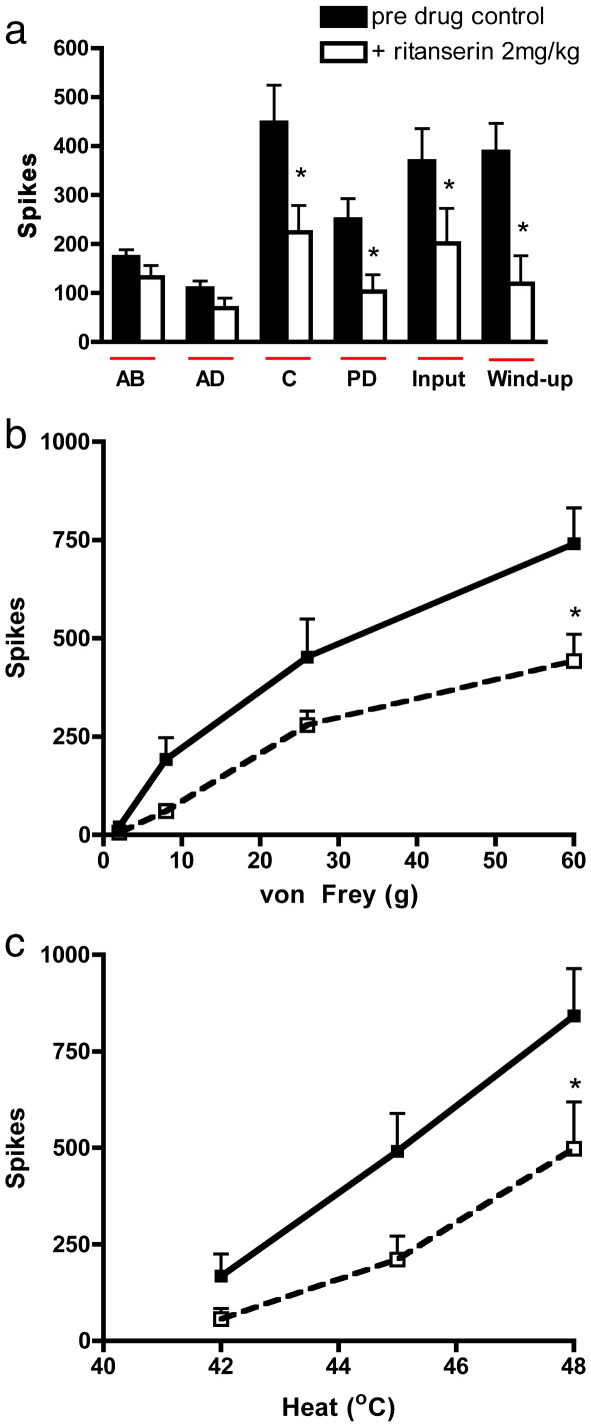
Effect of systemic administration of ritanserin (2 mg/kg) on a. electrical b. mechanical punctate and c. thermal evoked responses of wide dynamic range dorsal horn neurones. Ritanserin significantly inhibited the electrically evoked C-fibre, post discharge, input and wind-up neuronal responses. The evoked response to noxious mechanical (60 g) and heat (48 °C) stimuli was also significantly inhibited. Data expressed as mean ± SEM. Significance is denoted by *p < 0.05.

Ritanserin (2 mg/kg) significantly inhibited only the nociceptive specific elements of the electrical evoked neuronal response. This was seen as marked reductions in the evoked response to the C-fibre, post discharge, input and wind-up (*p* < 0.05, paired *t*-test) (Fig. 2a).

An overall inhibition of the natural mechanical and thermal evoked responses were observed following systemic ritanserin administration compared with pre-drug baseline control responses. Significance was seen in response to stimulation with vF 60 g and 48 °C heat (*p* < 0.05 2-way RM ANOVA). Although ritanserin clearly reduced the responses to the lower von Frey and heat stimuli tested, these did not quite reach significance (Fig. 2b and c). Interestingly, systemic administration of ritanserin produced near identical inhibitions to those seen with ketanserin with respect to the mechanical and thermal evoked neuronal responses, although the former drug produced greater inhibitions of the noxious electrical evoked responses (C-fibre, post discharge, input and wind-up) (Fig. 2a), as compared with the effects observed with ketanserin on the same electrical measures (Fig. 1a).

### 2.3 Effect of topical spinal administration of (±)-2,5-Dimethoxy-4-iodoamphetamine hydrochloride (DOI) on evoked dorsal horn neuronal responses

**Fig. 3:**
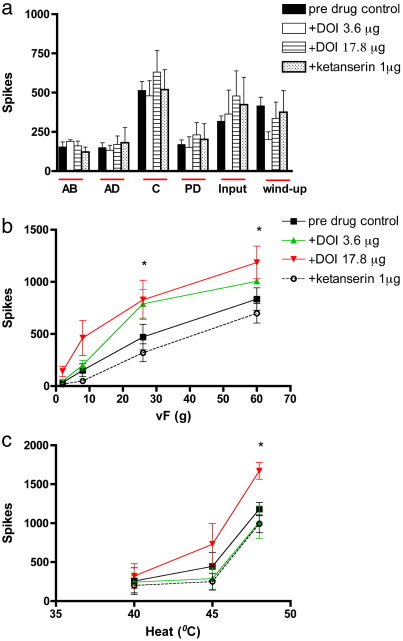
Effect of topical spinal administration of DOI (3.6 and 17.8 μg/50 μl) on a. electrical b. mechanical punctate and c. thermal evoked responses of wide dynamic range dorsal horn neurones. DOI significantly increased the evoked response to mechanical (26 and 60 g) and heat (48 °C) stimuli. Data expressed as mean ± SEM. Significance is denoted by *p < 0.05.

Spinal application of DOI did not produce any significant overall change in the electrical evoked neuronal responses (Fig. 3a). A trend towards a facilitation of the electrical C-fibre, post-discharge and input evoked neuronal responses was seen, but these effects did not reach statistical significance. In comparison the wind-up response tended to be inhibited by DOI. This effect may be partly due to the DOI induced increase in the input response. Wind-up is calculated as the number of “extra” neuronal responses evoked from a train of 16 electrical pulses after subtraction of the input response. Given that the input response tended to be facilitated by DOI, this resulted in a lowered wind-up value. This also suggests that DOI is more likely to have presynaptic site of action since the input gives a measure of the baseline C-fibre afferent input to the spinal cord prior to any spinal or supraspinal modulation of neuronal responses.

Spinal application of DOI resulted in an overall increase in the evoked neuronal responses to mechanical punctate stimuli, seen as a significant increase in evoked neuronal response to stimulation with von Frey 26 and 60 g (significant at 17.8 μg, *p* < 0.05 2-way RM ANOVA) (Fig. 3b). The DOI induced increase in mechanical evoked neuronal response was reversed back towards baseline levels by spinal application of ketanserin (1 μg).

The effect of DOI on the thermal evoked responses was more variable. Spinal application of DOI on the evoked response to 40 °C and 45 °C heat in 2 of the 6 cells resulted in a clear and sustained inhibition with one or both doses of DOI. Furthermore, in some instances, a transitory reduction was seen at the early (10 min.) time point to the evoked response to 40 °C and 45 °C stimuli; these inhibitory effects of DOI on these thermal stimuli were dwarfed by a marked facilitation of the neuronal response at the later time points (30 and 50 min.) post DOI administration. By contrast the evoked neuronal response to 48 °C was clearly facilitated (significant at 17.8 μg, *p* < 0.05 2-way ANOVA). The increased heat evoked neuronal responses produced by DOI were reversed back towards baseline levels by spinal application of ketanserin (Fig. 3c).

## 3 Discussion

There is considerable evidence for the critical role for serotonin (5-HT) in the modulation of spinal nociceptive transmission. A number of early studies observed inhibition and subsequent analgesia following blockade of the 5-HT system; more recently, however, a pronociceptive/hyperalgesic action has also emerged for the 5-HT system (for review see Bannister et al. (2009)). These contrary reports can, in part, be accounted for by the multiplicity of neuronal targets and receptor subtypes upon which 5-HT acts (Knight et al., 2004).

To date, seven distinct families of 5-HT receptors have been identified (5-HT1–5-HT7), and several of these have been further sub-classified. Among them, the 5-HT2 receptor is thought to play an important role in spinal pain modulation; however, as is the case for other 5-HT receptor subtypes, opposing data exist as to the direction of effect (pro- or antinociceptive) produced by 5-HT2 receptor modulation. Our electrophysiological data show that the 5-HT2A antagonist, ketanserin (10–100 μg/50 μl), and the 5-HT2A/2C antagonist, ritanserin (2 mg/kg), at these doses, have an overall inhibitory effect on spinal neuronal activity with selectivity for the higher intensity responses; furthermore, the 5-HT2A/2C receptor agonist, DOI, produced an overall facilitation of spinal neuronal responses with significant effects seen on the mechanical and thermal evoked neuronal responses. These increased neuronal responses were reversed by spinal application of ketanserin. Thus, our data support a pronociceptive role for the 5-HT2 receptor, most likely through targeting the 5-HT2A receptor subtype, on spinal nociceptive transmission under normal conditions.

There are several lines of evidence for a pronociceptive role for the 5-HT2 receptor, which is likely due to activity at the 5-HT2A receptor subtype. The 5-HT2A receptor has been shown to be widely distributed throughout the spinal cord and is present at presynaptic and postsynaptic sites therein. This receptor subtype shows dense labelling in lamina II inner and is therefore ideally located for modulation of spinal nociceptive processing. With regards to primary afferent neurones, 5-HT2A receptors are mainly localised in small and medium sized DRG neurones with most 5-HT2A receptor immunolabeled cells expressing the TRPV1 receptor, thus indicating their nociceptive nature (Doly et al., 2004; Van Steenwinckel et al., 2009). It is a G-protein coupled receptor positively coupled to phospholipase C, leading to an increase in phosphotidylinositol and intracellular calcium. *In vitro* electrophysiological recordings have shown a long lasting synaptic facilitation of superficial dorsal horn neurones mediated by 5-HT acting at 5-HT2 receptors (Hori et al., 1996). Taken together, these data would implicate an excitatory role for the 5-HT2A receptor in spinal nociceptive transmission.

The findings from behavioural studies are mixed. For instance, spinal administration of the mixed 5-HT2A/C agonist, (±)-2,5-dimethoxy-4-iodoamphetamine, (DOI), increased the behavioural response to formalin injection, an effect reversed by ketanserin (Kjorsvik et al., 2001), and DOI induced pain-like behaviours such as licking and biting, in line with a pronociceptive role for 5-HT2 receptors (Eide and Hole, 1991). Similarly, blocking spinal 5-HT2A receptors inhibited the formalin response (Nishiyama, 2005) and reduced spinal FOS activation in a paw incision model (Silveira et al., 2010). In direct contrast to the aforementioned studies, intrathecal administration of 5-HT2A/2C receptor agonists reversed the behavioural pain-like responses to formalin and chronic constriction nerve injury. These effects were reversed by pretreatment with intrathecal administration of ketanserin, therefore implicating spinal 5-HT2A receptors in mediating the antinociceptive effects of 5-HT (Sasaki et al., 2001; Sasaki et al., 2003), and 5-HT2A receptor induced spinal acetylcholine release and consequent antinociception was demonstrated (Kommalage and Hoglund, 2005) In rat models of chemotherapy and HIV-therapy induced neuropathy, however, a significant increase in 5-HT2AR immunoreactivity was seen in the superficial layers of the lumbar dorsal horn and an epidural injection of a selective 5-HT2A receptor antagonist dose-dependently decreased the thermal and mechanical hypersensitive behaviours; furthermore 5-HT2A receptor knockout mice did not develop HIV-therapy or chemotherapy-induced neuropathic pain behaviours whereas control littermates displayed a neuropathy comparable to that observed in rats (Thibault et al., 2008). By contrast, in a partial sciatic nerve ligation mouse model of neuropathic pain the inhibitory antiallodynic effects of a selective serotonin reuptake inhibitor was reversed by ketanserin (Honda et al., 2006). These discrepancies within the literature may be due to differences in the pain test or animal species used and also due to the inability of ligands used in earlier studies to sufficiently discriminate between 5-HT2A and 5-HT2C receptors.

The 5-HT2C receptor is present in the dorsal horn of the spinal cord, with 5-HT2C receptor mRNA expressed at high levels in most of the grey matter, except for lamina II (Fonseca et al., 2001). This receptor subtype is likely to have a predominant postsynaptic localization, since 5-HT2C mRNA was undetected in naïve rat DRG (Nicholson et al., 2003; Wu et al., 2001) but are expressed *de novo* in rat DRG after inflammation (Wu et al., 2001). The 5-HT2C receptor shares similar pharmacological and transductional features with the 5-HT2A receptor; however, with regards to modulation of spinal nociceptive transmission, a number of recent studies have assigned an antinociceptive role for this receptor subtype. For example, intrathecal administration of selective 5-HT2C receptor agonists attenuated pain-related behaviour in a rat model of trigeminal and spinal nerve ligated model of neuropathic pain, which may involve activation of spinal noradrenergic mechanisms (Nakai et al., 2010; Obata et al., 2004; Obata et al., 2007). Activation of spinal 5-HT2C receptors was also shown to reduce the C-fibre evoked spinal field potentials in spinal nerve ligated and sham control rats (Aira et al., 2010), and the selective 5-HT2C receptor antagonist RS 102221 reversed the inhibitory effect of spinal 5-HT on the evoked response of dorsal horn wide dynamic range neurons (Liu et al., 2007).

The 5-HT2 receptors have a very high amino-acid sequence homology and thus many compounds have an affinity for all three subtypes. Despite the selectivity limitations of drugs targeting 5-HT2 receptors, the emerging consensus, from the studies discussed above, points to a pronociceptive role for the 5-HT2A (Eide and Hole, 1991; Kjorsvik et al., 2001; Nishiyama, 2005; Silveira et al., 2010; Thibault et al., 2008) but see (Honda et al., 2006; Sasaki et al., 2001; Sasaki et al., 2003) and an antinociceptive role for the 5-HT2C receptor subtypes in modulating spinal nociceptive transmission (Aira et al., 2010; Liu et al., 2007; Obata et al., 2004; Obata et al., 2007).

Our findings in the present study demonstrate a clear pronociceptive role for spinal 5-HT2 receptors, most likely through targeting the 5-HT2A receptor subtype since the selective 5-HT2A antagonist ketanserin inhibited evoked neuronal responses, and in particular, inhibited the noxious evoked natural mechanical and thermal stimuli. Although ketanserin is the prototypical antagonist for 5-HT2A receptors, it also has affinity, but at higher concentrations, for the 5-HT2C receptor. Therefore, we cannot exclude the possibility that some of ketanserin's effect could be due to the blockade of 5-HT acting at 5-HT2C receptors.

Ritanserin has almost equal affinity for the 5-HT2A and the (reportedly antinociceptive) 5-HT2C receptor. Nonetheless, the overall effect of the drug was to reduce neuronal activity. Ritanserin produced significant inhibition of the electrically evoked, C-fibre, post discharge, input and wind-up, neuronal responses, in contrast to ketanserin, where no significant effect was seen on these electrically evoked neuronal measures. Both inhibited naturally evoked activity. Since we used naïve animals with no peripheral inflammation, it is unlikely that a peripheral action of ritanserin could be responsible. The difference could be due to a more potent and/or central effect of ritanserin or actions at supraspinal sites. For instance, 5-HT2A and 2C receptors are expressed within brainstem nuclei involved in descending pain modulation, e.g., RVM (Fonseca et al., 2001). However, the receptor here appears to produce an overall decrease in inhibitory outflow from descending pathways (de Oliveira et al., 2006; Kiefel et al., 1992; Queree et al., 2009), and these studies would predict that ritanserin effect within brainstem nuclei would increase spinal neuronal activity. However, there is some evidence for an excitatory response of medullary neurones to 5-HT, which is blocked by ketanserin (Davie et al., 1988); thus, it is conceivable that the dose of ritanserin used in our study could inhibit those neurones within the RVM classified as “ON cells” and which are deemed pain facilitating (Heinricher et al., 2009) so explaining the differences observed between local and systemic administration of the 5-HT2 antagonists.

Remarkably, ritanserin produced near identical inhibitory effects of the mechanical and thermal evoked responses as those seen with the top dose of spinal ketanserin, suggesting that the route of administration is not a critical factor in the overall effect of these two antagonists on naturally evoked neuronal activity and that the spinal cord is an important site of action of 5-HT2 receptor mediated pain facilitation.

DOI is a mixed 5-HT2A/2C receptor agonist, yet spinal application of the drug produced an overall increase in the evoked responses of spinal neurones to mechanical punctate and thermal stimulation of the peripheral receptive field, an effect that was reversed by ketanserin. Sasaki et al. (2001 and 2003) demonstrated an antinociceptive effect of DOI on behavioural responses in models of acute and sustained pain states; however, these studies used much higher doses of DOI. We have used lower doses of DOI, which are of a similar concentration with the doses used in studies demonstrating a pain-like behavioural syndrome induced by DOI (Eide and Hole, 1991; Kjorsvik et al., 2001). Thus, our data with the 5-HT2A/2C agonist, DOI, also support the conclusion that 5-HT2 receptors play a pronociceptive role in the spinal nociceptive transmission, possibly due to a predominant action of DOI at 5-HT2A receptors.

The overall effect on spinal neuronal activity is dependent on the interplay between excitatory and inhibitory mechanisms; thus, our data suggest that the overriding effects of ketanserin and ritanserin were likely to be mediated through antagonism of the actions of 5-HT acting at 5-HT2A receptors leading to the reduction in neuronal responses observed in this study. The consequence of 5-HT2C receptor blockade, at these doses, on the evoked spinal neuronal responses is minimal by comparison, if 5-HT2C receptors do indeed have an antinociceptive role. Similarly, activation of 5-HT2A/2C receptors with DOI increased neuronal responses, an effect reversed by ketanserin, thus implicating a predominant 5-HT2A action. An alternative possibility, however, is that 5-HT2C receptors could also have pronociceptive effect on spinal nociceptive transmission.

The primary source of descending serotonergic modulation of ascending nociceptive transmission from the spinal cord arises from the RVM (Millan, 2002). These serotonergic neurones can exert facilitatory or inhibitory influences onto dorsal horn neurones depending on the spinal 5-HT receptor subtype activated and the neuronal cell type within the RVM (Millan, 2002). Neurones within the RVM are classified into three types based upon their firing patterns in response to noxious thermal stimuli. ON-cells increase their firing immediately before a nocifensive response and facilitate nociception, while OFF-cells, considered to mediate inhibition, pause in their firing just prior to a nociceptive withdrawal reflex. Neutral cells do not appear to play a role in physiological pain (Heinricher et al., 2009).

Descending facilitation requires the activation of pronociceptive ON cells (Porreca et al., 2001); however, the pharmacology of descending facilitatory pathways remains unclear, as recordings from RVM neurones suggests that 5-HT containing neurones are neither ON nor OFF cells (Gao and Mason, 2000). However, converging evidence from recent immunohistochemical, behavioural and electrophyisological data suggests that a proportion of RVM cells activated by noxious stimuli are serotonergic. Furthermore, a facilitatory effect mediated by 5-HT, acting at spinal 5-HT3 receptors, was demonstrated in models of acute and chronic pain (Oatway et al., 2004; Rahman et al., 2004; Rahman et al., 2006; Suzuki et al., 2002; Suzuki et al., 2005; Svensson et al., 2006). These studies focused on the pronociceptive 5-HT3 receptor, the only ligand gated cation channel of the 5-HT receptor family, and its role in mediating descending facilitation.

The electrophysiological consequences of selectively blocking spinal 5-HT2A receptors on dorsal horn neuronal activity are similar to the effects we have previously seen with the selective 5-HT3R antagonist ondansetron (Suzuki et al., 2002). In the present study, the top dose of ketanserin (100 μg) significantly inhibited the evoked responses to vF 26 and 60 g and to 45 and 48 °C heat stimuli, we have previously shown that ondansetron (100 μg) also reduced the evoked neuronal responses to these stimuli to a similar degree (Rahman et al., 2004; Suzuki et al., 2002). Therefore, descending serotonergic facilitation could equally be mediated through 5-HT acting at spinal 5-HT2A receptors.

## 4 Conclusion

The present study provides electrophysiological evidence for a pronociceptive role for spinal 5-HT2 receptors on the evoked responses of deep dorsal horn wide dynamic range neurones and supports a pronociceptive role for the 5-HT2A receptor on spinal nociceptive transmission, without excluding a 5-HT2C involvement. The data also implicate a role for 5-HT2 receptors in mediating descending facilitation of spinal nociceptive processing.

Interestingly, evidence from pain patients suggests that 5-HT2A receptor gene polymorphisms could influence individual differences in pain sensitivity (Bondy et al., 1999; Pata et al., 2004) and a recent study has demonstrated a link between single nucleotide polymorphisms in the 5-HT2A receptor gene and individual analgesic requirements for post-operative pain management (Aoki et al., 2010). Therefore, unravelling the role of the 5-HT2A receptor in pain modulation presents a promising avenue for future drug development and pain therapy.

## 5 Experimental procedures

Male Sprague–Dawley rats (230–250 g) were employed for this study (Central Biological Services, University College London, UK). All experimental procedures follow the UK Animals (Scientific Procedures) Act 1986 and the guidelines under the International Association for the Study of Pain (Zimmermann, 1983).

### 5.1 Electrophysiology

Animals were anaesthetised with isofluorane (1.5–1.7%; 66% N_2_O and 33% O_2_) and a laminectomy was performed to expose the L4-5 segments of the spinal cord. Extracellular recordings were made from ipsilateral deep dorsal horn neurones (lamina V–VI) using parylene coated tungsten electrodes (A-M Systems, USA). A train of 16 transcutaneous electrical stimuli (2 ms wide pulses, 0.5 Hz) was applied at 3 times the threshold current for C-fibres; following which a post-stimulus histogram was constructed. Responses evoked by Aβ-(0–20 ms), Aδ- (20–90 ms) and C-fibres (90–350 ms) were separated and quantified on the basis of latency. Neuronal responses occurring after the C-fibre latency band of the neurone were classed as post-discharge, a result of repeated stimulation leading to wind-up neuronal hyperexcitability. The ‘input’ (non-potentiated response), and the ‘wind-up’ (potentiated response, evident by increased neuronal excitability to repeated stimulation) were calculated. Input = (action potentials evoked by first pulse at 3 times C-fibre threshold) × total number of pulses (16). Wind-up = (total action potentials after 16 train stimulus at 3 time C-fibre threshold) − Input. The peripheral receptive field was also stimulated using a range of natural stimuli (von Frey filaments, 2, 8, 26 and 60 g and heat, 42, 45 and 48 °C) applied over a period of 10 s per stimulus and the evoked response quantified. The heat stimulus was applied with a constant water jet onto the centre of the receptive field. Data were captured and analysed by a CED 1401 interface coupled to a Pentium computer with Spike 2 software (Cambridge Electronic Design; PSTH and rate functions).

### 5.2 Drug administration

Stable control responses to electrical and selected natural stimuli were established at 20 min intervals prior to drug administration; this was confirmed with at least 3 consistent responses (< 10%) to all measures. Means of these baseline responses were calculated and used as the ‘pre-drug’ controls from which drug effects on subsequent evoked responses were tested against. Ketanserin (1, 10 and 100 μg/50 μl saline) was applied topically to the spinal cord in a cumulative manner or ritanserin (2 mg/kg) was administered subcutaneously into the scruff of the neck. DOI (3.6 and 17.8 μg/50 μl saline) was applied topically to the spinal cord in a cumulative manner; a low dose of ketanserin (1 μg/50 μl/saline), which does not produce any effect on neuronal activity on its own, was then administered to the spinal cord. The two routes were used because spinal application of a drug will localise its pharmacological target to pre-or postsynaptic elements in the dorsal horn. We used sub cutaneous administration to assess the effects of systemic exposure. The effect of each drug was followed over an hour per dose, with tests carried out at 10, 30 and 50 min time points post drug application. The nature of the drug injection and recording protocol meant that just one experiment, on one neurone, was performed per animal used.

### 5.3 Data analysis

Data are presented as mean ± standard error of mean (SEM) unless otherwise stated. For all studies the maximal effect, compared with pre-drug baseline control, for each dose was selected, this varied and was seen at any of the time points tested i.e. 10, 30 and 50 min post drug administration. However, in most cases the maximal change in response was observed at 30 or 50 min post drug application. Drug effects were then expressed as the mean maximal effect of the pre-drug control for each dose. Analyses were performed using GraphPad Prism version 4 for Apple Macintosh OS 10.4, (GraphPad Software, USA), and for all data, a 95% confidence interval was used as a measure of statistical significance. All statistical analyses were performed on raw data using two-way analysis of variance with repeated measures (RM ANOVA) for responses to mechanical and thermal stimuli, and if significant, Bonferroni post hoc tests were performed. The effect of ketanserin and DOI effect on responses to electrical stimulation were assessed using a one-way RM ANOVA followed by Dunnett's post hoc multiple comparisons test for significant values. The effect of ritanserin on electrical evoked responses was assessed using a paired Student's *t*-test.
